# The Antibiofilm efficacy of nitric oxide on soft contact lenses

**DOI:** 10.1186/s12886-017-0604-2

**Published:** 2017-11-21

**Authors:** Dong Ju Kim, Joo-Hee Park, Marth Kim, Choul Yong Park

**Affiliations:** Department of Ophthalmology, Dongguk University, Ilsan Hospital, 814, Siksadong, Ilsan-dong-gu, Goyang, Gyunggido 410-773 South Korea

**Keywords:** Nitrite, Nitric oxide, Biofilm, Contact lens, Bacterial keratitis, Cornea

## Abstract

**Background:**

To investigate the antibiofilm efficacy of nitric oxide (NO) on soft contact lenses.

**Methods:**

Nitrite (NO precursor) release from various concentrations (0–1000 μM) of sodium nitrite (NaNO_2,_ NO donor) was measured by Griess Assay. Cell viability assay was performed using human corneal epithelial cell under various concentration (0–1000 μM) of NaNO_2_. Biofilm formation on soft contact lenses was achieved by adding *Staphylococcus aureus* or *Pseudomonas aeruginosa* to the culture media. Various concentrations of NaNO_2_ (0–1000 μM) were added to the culture media, each containing soft contact lens. After incubation in NaNO_2_ containing culture media for 1, 3, or 7 days, each contact lens was transferred to a fresh, bacteria-free media without NaNO_2_. The bacteria in the biofilm were dispersed in the culture media for planktonic growth. After reculturing the lenses in the fresh media for 24 h, optical density (OD) of media was measured at 600 nm and colony forming unit (CFU) was counted by spreading media on tryptic soy agar plate for additional 18 h.

**Results:**

Nitrite release from NaNO_2_ showed dose-dependent suppressive effect on biofilm formation. Most nitrite release from NaNO_2_ tended to occur within 30 min. The viability of human corneal epithelial cells was well maintained at tested NaNO_2_ concentrations. The bacterial CFU and OD showed dose-dependent decrease in the NaNO_2_ treated samples on days 1, 3 and 7 for both *Staphylococcus aureus* and *Pseudomonas aeruginosa*.

**Conclusions:**

NO successfully inhibited the biofilm formation by *Staphylococcus aureus* or *Pseudomonas aeruginosa* on soft contact lenses in dose-dependent manner.

## Background

Bacteria accounts for most cases of the infectious keratitis. If not properly treated, bacterial keratitis can cause sight-threatening complications, such as corneal opacity or perforation [1–3]. Contact lens wearing significantly increases the risk of bacterial keratitis and it was reported that about 3.5 to 20 soft contact lens wearers developed bacterial keratitis every year [4]. Corneal hypoxia and epithelial damage induced by wearing contact lenses enhances the bacterial adhesion and invasion [4]. Normal host defense mechanism against pathogen, such as tear and blinking can also be significantly interfered by wearing contact lenses [5]. Furthermore, mucins and proteins can attach on the surface of contact lens and promote the pathogen adhesion. The adhesion of specific bacteria to contact lens can form a biofilm [5]. A biofilm is a complex of microbial communities enclosed in an exopolysaccharide matrix adhered to the surface of prosthetics or living organism [6, 7]. Biofilms enable bacteria to survive in unfavorable environments by reducing their metabolic needs, inhibiting the penetration of antimicrobial agents and increasing their inherent resistance to antimicrobial agents [8]. In addition, biofilms formed on contact lenses can play a critical role in developing bacterial keratitis as a depot for continuous bacterial release [8–10].

Nitric oxide (NO) is a one of well-known anti-bacterial mechanisms of mammalian host [11]. As a small molecular gas, NO can diffuse freely across the cellular membrane [12]. When generated locally with micromolar concentration, NO acts as a cytotoxic antimicrobial agent [11]. In addition, previous studies have shown that NO could act as a key mediator for biofilm dispersal [13, 14]. It was reported that low concentrations of NO induced biofilms dispersal while high concentrations of NO directly killed pathogens [15]. It is known that high concentrations of NO have a broad antibiotic spectrum that can act on both gram positive and negative or both mono-species and multi-species biofilms [16–20].

Bacteria such as *Staphylococcus aureus and Pseudomonas aeruginosa* contains nitrite reductase which converts nitrite (NO_2_^−^) to NO. The gene encoding nitrite reductase is known as *nas*DE or *nir*BD in *Staphylococcus aureus* and *nir*S in *Pseudomonas aeruginosa* [21, 22].

Although the evidences of anti-biofilm effect of NO are growing, the study investigating the effect of NO on soft contact lens-associated biofilms is hard to find. Considering the clinical importance of contact lens associated biofilms and resulting bacterial keratitis, the role of NO on soft contact lens-associated biofilm is an interesting research topic.

In the current study, we used sodium nitrite (NaNO_2_) as a NO donor and investigated the inhibitory effects of NO on soft contact lens-associated biofilms by *Staphylococcus aureus and Pseudomonas aeruginosa*, which are two of most common strains of infectious keratitis [23, 24].

## Methods

### Griess assay for nitrite release

The nitrite release from NaNO_2_ (Sigma_−_Aldrich St. Louis, MO, USA) was measured using Griess Reagent System Kit (catalog number: G2930; Promega Corp., Madison, WI, USA). The Griess Reagent System is based on the chemical reaction, which uses sulfanilamide and N-1-napthylethylenediamine dihydrochloride (NED) under acidic conditions. This system detects nitrite in liquid matrices. A nitrite standard reference curve was obtained for the accurate quantitation of nitrite levels in experimental samples. A sulfanilamide solution (50 μL) was dispensed to all experimental samples, which were then incubated for 10 min at room temperature while protected from light. After the incubation, 50 μL of the NED solution was added to all samples, which were then further incubated at room temperature for 10 min with light protection. Finally, the measurement of absorbance at 540 nm was performed with a plate reader. The nitrite release from NaNO_2_ at different concentrations (0, 0.1, 1, 10, 100, 1000 μM) was tested for 30 m, 6 h and 24 h.

### Cell viability assay

Cell viability assay was performed using CCK-8 reagent (Dojindo Molecular Technologies, Inc. Kumamoto, Japan) according to the manufacturer’s protocol. Briefly, human corneal epithelial cells (HCECs) were cultured at 4 × 10^3^ cells/ well in a 96-well plate and incubated for 24 h. Following the adherence of cells, NaNO_2_ was dose dependently treated to cells for 3 h, 6 h, 24 h, and 48 h at concentrations of 0, 0.1, 1, 10, 100 and 1000 μM. After the appropriate incubation, 10% (*v*/v) of CCK-8 solution was added to the culture media and the absorbance at 450 nm was measured after 2 h of incubating the HCECs with the reagent.

### Bacterial culture

*Staphylococcus aureus* (*S. aureus*; ATCC 25923) and *Pseudomonas aeruginosa* (*P. aeruginosa*; ATCC 10145) were purchased from American type culture collection (ATCC; Manassas, VA, USA). Both bacteria were cultured in a tryptic soy broth (soybean-casein digest media; Becton Dickinson and Company; BD, Franklin Lakes, NJ, USA) and were maintained at 37 °C incubator under aerobic condition with soft contact lenses (Hioxifilcon-A, Interojo, Gyeonggi-do, Pyeongtaek, South Korea).

### Treatment of NaNO_2_ in bacterial culture

**Fig. 1:**
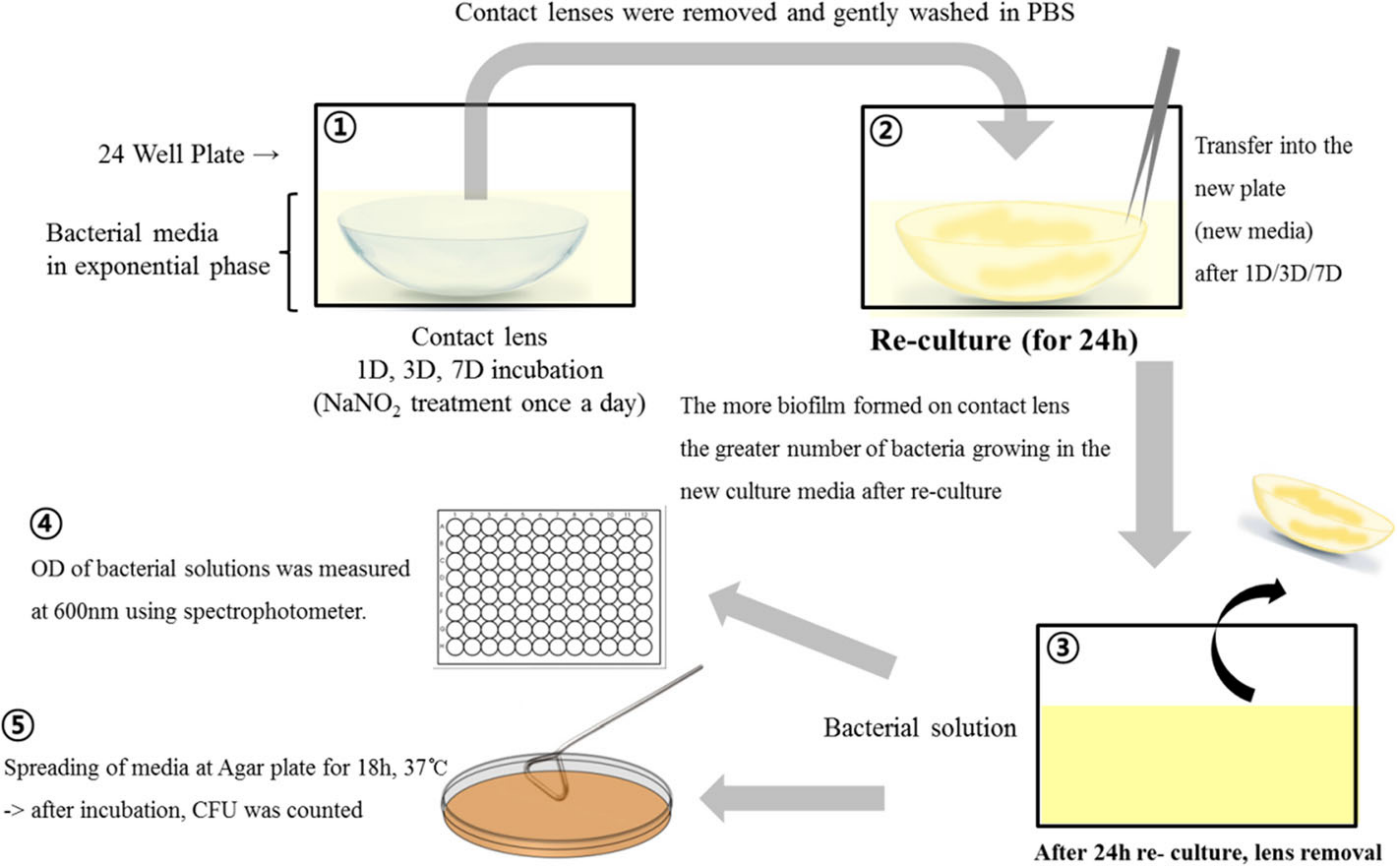
Schematic illustration showing the reculturing methods used to evaluate antibiofilm effect of nitric oxide. *1)* Soft contact lens was incubated with bacteria for 1–7 days. *2)* To analyse the biofilm attached to each contact lens, only the contact lens was harvested and transferred to fresh culture media for additional 24-h culture. *3*, 4 & 5 *)* After 24 h of re-culture, the bacterial burden dispersed in the culture media was analysed by optical density measurement and colony forming unit counting

Soft contact lenses and bacteria (*S. aureus* or *P. aeruginosa*) were cultured together with the treatment of various concentrations (0, 0.1, 1, 10, 100 and 1000 μM) of NaNO_2_. The bacteria were cultured 24 h in 24-well plate before the treatment of NaNO_2_ and was used at ≥ 0.55 (optical density (OD)_600_). A stock solution of 1 M–NaNO_2_ were serially diluted and gently mixed with culture media to reach the final desired concentrations of NaNO_2_ and the media were maintained at 37 °C in an incubator under aerobic condition. NaNO_2_ was mixed to the culture media once a day. On first, third, and seventh days, cultured soft contact lenses were removed from the plate and gently washed in phosphate buffered saline(PBS) for three times, and then transferred to new 24-well plate containing fresh media for reculturing for additional 24 h. After 24 h of reculturing, the optical density (OD) of bacterial solutions was measured at wavelength of 600 nm using a spectrophotometer (SpectraMax plus 384 microplate reader, Molecular Devices, Sunnyvale, Ca, USA) (Fig. 1).

### Colony forming unit (CFU) assay

The media 24 h after the reculturing of the contact lenses were diluted and spread on a tryptic soy agar (Becton Dickinson and Company, Franklin Lakes, NJ, USA) plate, and maintained at 37 °C in an incubator under aerobic conditions for another 18 h. After the culturing, the bacterial colonies were counted (Fig. 1).

### Statistical analysis

The experiments were conducted three times in total, and the results were derived from all three sets of experimental data. IBM SPSS ver. 20.0 (IBM Corp., Armonk, NY, USA) was used for all the statistical analyses. The statistically significance of CFU, OD, cell viability was determined using one-way analysis of variance (ANOVA) followed by the Tukey honestly significant difference (HSD) test. *P* values less than 0.05 were considered statistically significant.

## Results

### Griess assay for measuring released nitrite

**Fig. 2:**
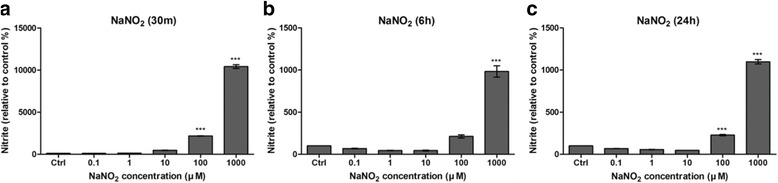
Nitrite release measured by Griess assay. Nitrite was released dose dependently from NaNO_2._ As shown, most nitrite release from NaNO_2_ was achieved within 30 min (**a**). Nitrite release significantly decreased to less than 10% when measured after 6 (**b**) and 24 h (**c**). Due to the sensitivity of the assay, significant nitrite release was detectable with only high concentrations (100 or 1000 μM) of NaNO_2_

Most nitrite release from NaNO_2_ occurred within 30 min, as shown in Fig. 2. Nitrite release decreased significantly as time elapsed, reaching approximately 10% of the initial nitrite release when measured after 6 h.

### Cell viability

**Fig. 3:**
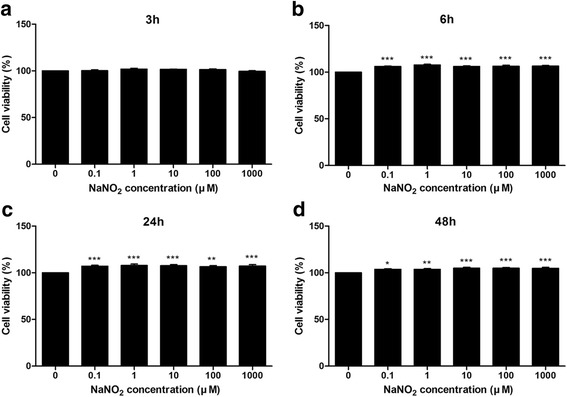
Human corneal epithelial cell viability assay. Human corneal epithelial cell viability was measured using CCK-8 kit after 3 (**a**), 6 (**b**), 24 (**c**) and 48 h (**d**) of incubation with various concentrations (0.1–1000 μM) of NaNO_2_. The graphs showed a mild increase in cell viability with NaNO_2_ addition at 6, 24 and 48 h. No detrimental effect of NaNO_2_ on cell viability was observed at the tested concentrations. Triplicates of each treatment group were used in each independent experiment. Values are the mean ± standard error of means from three independent experiments. (*p < 0.05, **p < 0.01, ***p < 0.001)

In general, no detrimental effect of NaNO_2_ on cellular viability was observed (Fig. 3). A mild increase in cellular viability was observed with 0.1–1000 μM of NaNO_2_ after 6, 12, and 24 h of incubation.

### Nitrite effect of contact lens biofilm

**Fig. 4:**
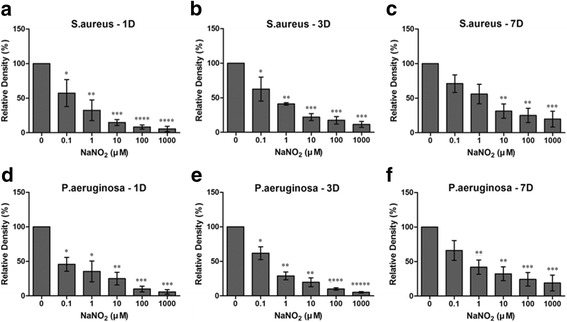
Nitric oxide’s effect on contact lens associated biofilm analyzed by optical density analysis. Optical density (OD) of samples was converted to relative density (%) (the OD of the sample divided by OD of the negative control) and was shown in bar graphs. **a**–**c** OD comparison in samples with *Staphylococcus aureus*. The OD was lower than the negative control at all concentrations of NaNO_2_ at days 1 and 3. The effect of NaNO_2_ on biofilm formation was dose dependent. At day 7, low concentrations (0.1 and 1 μM) of NaNO_2_ decreased the biofilm but failed to reach statistical significance. Higher concentrations (10–1000 μM) of NaNO_2_ significantly decreased the biofilm formation even at day 7. **d**–**f** OD comparison in samples with *Pseudomonas aeruginosa.* The OD was lower than the negative control at all concentrations of NaNO_2_ at days 1 and 3. The effect of NaNO_2_ on biofilm formation was dose dependent. At day 7, low concentrations (0.1 μM) of NaNO_2_ decreased the biofilm but failed to reach statistical significance. Higher concentrations (1–1000 μM) of NaNO_2_ significantly decreased the biofilm formation even at day 7. Statistical significance was determined using one-way analysis of variance (ANOVA) followed by the Tukey HSD test. *Statistically significantly higher than control group, **Statistically significantly higher than 0.1 μM group.***Statistically significantly higher than 1 μM group,****Statistically significantly higher than 10 μM group,*****Statistically significantly higher than 100 μM group

**Fig. 5:**
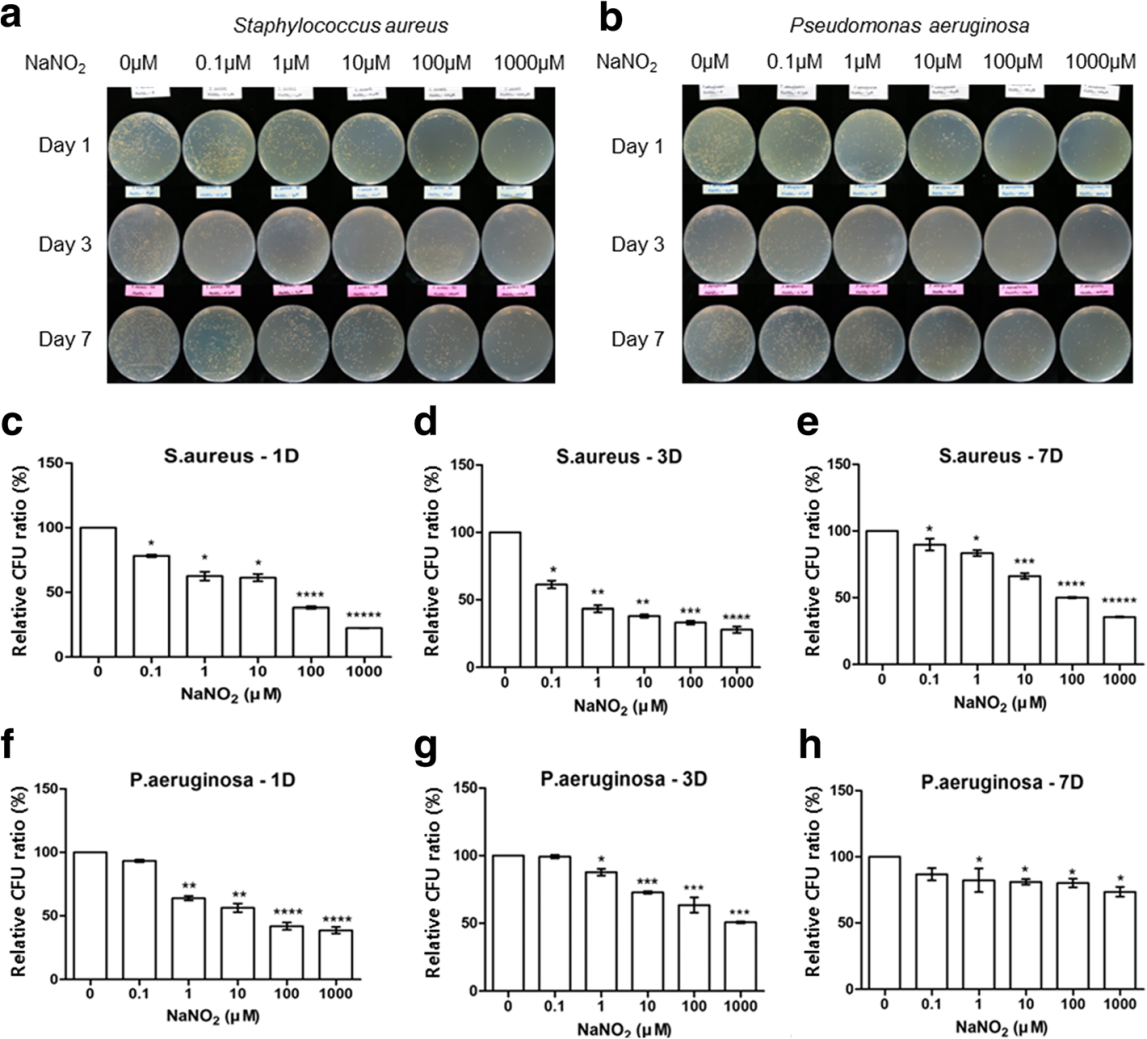
Nitric oxide’s effect on contact lens-associated biofilm analyzed by colony forming unit counting analysis. **a** and **b** Representative pictures of agar plates showing the colonies after 24 h of reculturing. Colony forming unit (CFU) count decreased with increasing concentrations of NaNO_2_ in both *S. aureus* and *P. aeruginosa.* The number of scattered colonies decreased with increasing concentrations of NaNO_2_. **c**–**h** The CFU count of the samples was converted to the ratio (%) (the CFU count of the sample divided by CFU count of the negative control) and was shown in bar graph. The CFU count of both *S. aureus* and *P. aeruginosa* shows dos-dependent decrease with increasing concentration of NaNO_2_. The suppressing effect of NaNO_2_ decreased at day 7 in biofilm formed by *P. aeruginosa.* Statistical significance was determined using one-way analysis of variance (ANOVA) followed by the Tukey HSD test. *Statistically significantly higher than control group, **Statistically significantly higher than 0.1 μM group.***Statistically significantly higher than 1 μM group,****Statistically significantly higher than 10 μM group,*****Statistically significantly higher than 100 μM group

NaNO_2_ decreased the biofilm formation of both *S. aureus* and *P. aeruginosa* in a dose-dependent manner (Figs. 4 and 5). Reculturing contact lenses with biofilm is a kind of indirect method for quantification of biofilm. Larger amounts of biofilm contain a heavier burden of pathogens. After change of the environment (from an NO positive to an NO negative culture condition), pathogens can release from the biofilm to culture media for planktonic growth. Exponential growth of pathogen can change the OD of the culture media, making the media more turbid. OD measured in this study therefore represent the pathogen burden contained in biofilm.

The OD of recultured media decreased with the addition of NaNO_2._ Even a low concentration (0.1 μM) of NaNO_2_ decreased the biofilm by almost 50% compared with the control. A high concentration (1 mM) of NaNO_2_ further decreased the biofilm formation to less than 10% of the control even on the first day (Fig. 4). Similar findings were repeated with CFU analysis. However, the effect of NaNO_2_ measured by CFU analysis seemed to be blunted compared to OD analysis at day 7, especially with *P. aeruginosa* (Fig. 5).

## Discussion

In this study, we found that NO showed a dose-dependent suppression of the biofilm formed on soft contact lenses by *S. aureus* and *P. aeruginosa*. In addition, the tested concentrations of NaNO_2_ showed no significant toxicity on cultured human corneal epithelial cells.

Biofilm formation on the surface of the medical device is closely related to various infectious diseases because it increases continuous pathogen dispersion [7, 8, 25]. It is known that biofilm forms commonly on contact lenses and is one of the major risk factors for infectious keratitis [1, 26–28]. The characteristics of the contact lenses, such as hydrophobicity or surface irregularity, can also affect bacterial attachment and subsequent biofilm formation [29]. Furthermore, biofilm formation on the anterior surface of contact lens can be somewhat prevented by natural host defense mechanisms such as blinking movement and antimicrobial components within tears [30]. However, the posterior surface of the contact lens touches the cornea and this contact interferes natural defense mechanisms [31]. Therefore, biofilm is found commonly on the posterior surface of contact lens [9]. Bacteria within biofilm are more resistant to antibiotics and host defense mechanisms compared to free planktonic form of bacteria [6, 32].

Previous studies found that NO is a key mediator of biofilm dispersal [13, 15, 16, 33, 34]. Biofilm dispersal needs some molecular triggers which induce a change from biofilm to dispersal phenotype. One of these triggers is a low concentration of NO and another is a decrease of intracellular c-di-GMP [17, 35, 36]. Low concentrations of NO induce biofilm dispersal via signal cascades involving both increase of phosphodiesterase activity and decrease of intracellular c-di-GMP [14, 15]. In addition, planktonic bacteria exposed to low concentrations of NO become even more susceptible to other antibiotics [37, 38].

It is known that high concentrations of NO are broad-spectrum bactericidal agents that can kill both gram positive and negative bacteria [38–40]. However, NO can also be toxic to host cells at high concentrations. High concentrations of NO can be converted to reactive nitrogen species such as peroxynitrite and reactive nitrogen species can cause cytotoxic effects not only on pathogens but also on host cells.

NO is an unstable free radical gas and has limited solubility in water [12]. This makes it difficult to introduce pure gaseous NO into the culture media. Therefore, various chemical agents that release NO, such as sodium nitroprusside, sodium nitrate (NaNO_3_) or sodium nitrite (NaNO_2_), have been widely used as NO donor for biological experiment evaluating NO effect [17, 41]. In our study, NaNO_2_ was used as a NO donor. NaNO_2_ yields nitrite and nitrite can be converted to NO by nitrite reductase. In the previous report, exogenous supply of NaNO_2_ successfully decreased the biofilm formation on the culture plate by *S. aureus* and *P. aeruginosa* [42].

We tested the safety of NaNO_2_ of concentrations (0.1, 1, 10, 100 and 1000 μM) using HCECs. The viability of HCECs was not interrupted by NaNO_2_ up to 48 h at all tested concentrations. In addition, the contact lens biofilm formed by *S. aureus* and *P. aeruginosa* successfully decreased following addition of NaNO_2_. Our results are consistent with previous studies that showed the suppressive effect of NO on biofilm and bacteria on other medical devices [13, 33, 34].

In this study, CFU count tended to increase over a week even with the presence of NO especially in *P. aeruginosa*. We hypothesize that biofilm formation was inhibited only during the short action period of NO and the residual bacteria then proliferated again. In our study, NaNO_2_ was applied once a day to avoid significant change of osmotic pressure of culture media. As a relatively unstable gas, NO has a short half- life [12]. As shown in Fig. 2, the most robust action of NO was expected within 30 min every day in our experimental setting. Therefore, the increase in the CFU count at day 7 might be the cumulative effect of bacterial regrowth during NO-free period of each day. Another possible explanation might be the limited diffusion of NO through the already established biofilm formation by *P. aeruginosa* at day 7. As previously known, biofilm can protect microorganisms by providing mechanical diffusion barrier to antimicrobial agent [8].

In previous reports, the optimal NO concentration for biofilm suppression was different in two bacteria studied. *P. aeruginosa* was more susceptible to biofilm dispersal at lower concentrations (0.025 ~ 2500 nM) of NO. [13, 33] On the other hand, relatively high concentrations (124 ~ 1000 μM) of NO was necessary to inhibit biofilm formation by *S. aureus* [43]. However, our result revealed that both bacteria were susceptible to biofilm dispersal at similar NaNO_2_ concentration range (0.1 to 1000 μM). We believe that the difference in the strains and experimental settings (biofilm formation on soft contact lens and different NO donor) may explain the discrepancies between studies.

Although it is one of the first pioneering study to evaluate NO effect on contact lens associated biofilm, this study has several limitations. The first, the interaction of bacteria and host immune system and its modulation by NO was not elucidated in our *in vitro* study. Secondly, we used the indirect method for the quantification of biofilm, the re-culturing from biofilm, instead of direct quantification of biofilm. We initially tried direct staining and biofilm quantification on contact lenses using crystal violet after complete drying of contact lens. However, there were several drawbacks that made us abandon the direct staining method. We found that the contact lens itself was heavily stained by crystal violet. In addition, a significant amount of biofilm formed on contact lens was not fixed and continuously dropped out during washing, drying and staining procedure. In addition, thermal fixation could not be applied because it would burn the contact lens. Therefore, we chose the indirect quantification method and assumed that only the biofilm attached to the contact lens was the source of bacterial growth in the recultured media. The similar methods were reported to quantify biofilm development on intraocular lens surface previously [44, 45]. In our study, both OD measurement and CFU count were adopted to increase the accuracy of biofilm quantification. Thirdly, NaNO_2_ was treated only once a day because osmotic change from the additional sodium in NaNO_2_ can affect bacterial growth. NO release from NaNO_2_ is expected to be quite concentrated within 30 min of NaNO_2_’s application to the culture media, so a more stable and continuous NO donor would be optimal for investigating dose-dependent effect of NO. Furthermore, the exact quantification of NO production from nitrite was not measured in this study. The difference between experimental and physiological conditions should be also considered for interpretation of our results. The dynamic tear clearance and lens movement were absent in our experimental settings and these two factors might change the biofilm kinetics in human ocular surface [46]. Finally, the effect of NO on multispecies biofilms was not investigated even though multispecies-associated keratitis is common with contact lens wear [27].

The clinical significance of our results is the potential use of NO as an active disinfectant in contact lens care solution. NO donors such as NaNO_2_ or sodium nitroprusside are already used as intravenous medications for the treatment of cyanide poisoning (NaNO_2_) and heart diseases (sodium nitroprusside) [47, 48]. Therefore, if ophthalmic topical safety is ensured through clinical trials, a tablet form of these chemicals can be mixed into daily dispense of contact lens care solution to eradicate bacterial biofilm.

## Conclusion

In conclusion, we confirmed that NO can suppress biofilm formation on soft contact lens in a dose dependent manner. Our findings suggest that NO can be developed as a new therapeutic strategy to reduce biofilm-associated contact lens infection with minimal toxicity to corneal epithelium.

## Abbreviations

CFU: Colony forming unit
NO: Nitric oxide
OD: Optical density
